# A bronchogenic cyst masquerading as asthma: A case report

**DOI:** 10.7196/SARJ.2018.v24i2.169

**Published:** 2018-06-21

**Authors:** M D Moremi, A L Motene, N J Maligavhada, N G Tiva, R T Mamogale, S M Risenga

**Affiliations:** 1 Department of Paediatric Pulmonology and Allergy, University of Limpopo and Pietersburg Provincial Hospital, Polokwane, South Africa; 2 Department of Diagnostic Radiology, University of Limpopo and Pietersburg Provincial Hospital, Polokwane, South Africa

**Keywords:** paediatric pulmonology, bronchogenic cyts, South Africa, paediatric male, case study

## Abstract

Wheezing in infants and under-five children may present a diagnostic problem as there are various aetiologies for this symptom. Diagnosis of asthma is often made as it is one of the causes of wheezing in children. It is however important to have taken a complete history, including allergy and appropriate diagnostic investigations. If the child’s symptoms do not improve despite appropriate therapy, a different diagnosis must be pursued. We report the case of a child who presented to us with wheezing and who did not respond to therapy.

## Background

Bronchogenic cysts (BCs) of the mediastinum are uncommon congenital malformations arising from the primitive foregut.^[1]^ Mediastinal congenital bronchogenic cyst, or those found anywhere in the body are usually asymptomatic.^[2]^ They are at times discovered as an incidental finding, becoming a source of diagnostic confusion. ^[2]^ Symptoms develop when they compress adjacent structures, become infected or increase in size.^[2]^ Most BCs are asymptomatic at birth.^[3]^ The majority of BCs are mediastinal and some can occur in the lung parenchyma.^[4]^

## Case

A 27-month-old boy was referred from one of our district hospitals to the Pietersburg tertiary hospital with an assessment of an acute asthmatic attack. The child was in severe respiratory distress and not responding to therapy, which included salbutamol nebulisations and oral prednisone. The child did not have a history of haemoptysis, fever, dysphagia, diarrhoea or weight loss. There was no history of tuberculosis contact in the household. He was fully immunised with normal milestones and good nutrition.

Previous admissions could not be elicited although he was treated on a few occasions by his general practitioner for recurrent chest infections and noisy breathing prior to this admission. The therapy at the time included salbutamol syrup, which is not standard therapy for asthma, oral prednisone and antibiotics; with no improvement.

Two months before this presentation he was diagnosed with asthma at a district hospital where he was treated with budesonide metred dose inhaler (MDI) 100 µg twice a day and 2 puffs of salbutamol MDI 100 µg when needed. However, there was minimal clinical response to this therapy, with persistence and worsening of symptoms.

Clinical examination on the day of admission revealed severe respiratory distress as evidenced by nasal alar flaring, intercostal and subcostal recessions. The child was not cyanosed but had signs of superior vena caval syndrome as evidenced by distended neck veins, cough, facial swelling and hoarseness of voice. He had no signs of chronic lung or cardiac disease but had significant lymphadenopathy (>2 cm) involving axillary and inguinal regions. He had audible wheezes, with tracheal tug and a hoover sign. His heart sounds were normal, with no signs of pulmonary hypertension. Vital data monitoring while on oxygen by face mask showed saturations of 80%, a respiratory rate of 67 breaths per minute and heart rate of 182 beats per minute.

The patient was intubated and ventilated in the paediatric intensive care unit (PICU) and commenced on oral prednisone and nebulised with fenoterol and ipratropium bromide hourly as a continuation of asthma therapy while looking for an alternative diagnosis. He was started on second-line antibiotics, tazocin and amikacin and required sedation with dormicum and morphine.

The initial assessment was that of an intrathoracic airway obstruction secondary to a mediastinal mass and a respiratory tract infection.

**Fig. 1:**
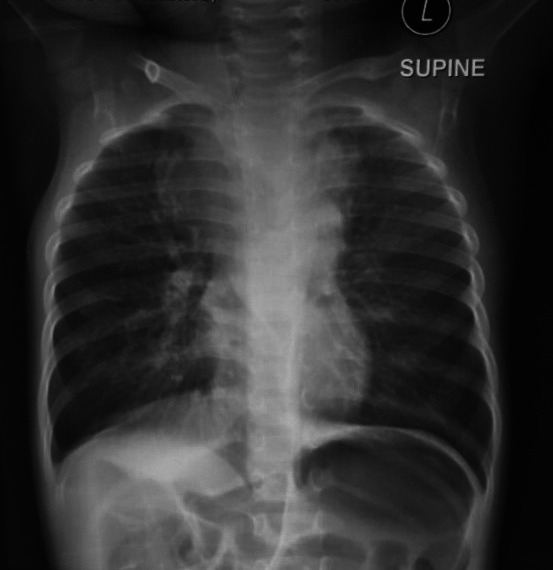
Pre-operative chest X-rays of the patient (not intubated). Widening of the superior mediastinum with early left lower-lobe patchy infiltrates and right lung upper-lobe early herniation to the left.

**Fig. 2:**
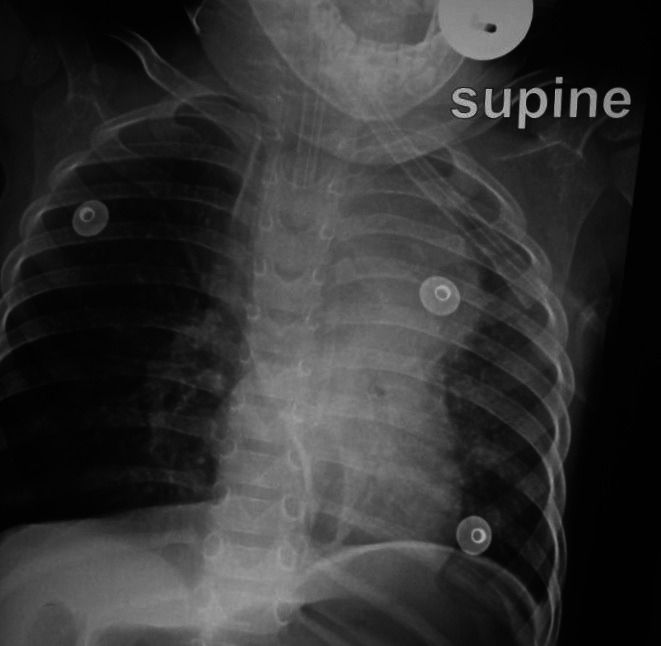
Patient intubated, rotated, left-lung early collapse consolidation and middle lobe consolidation.

The following investigations were performed:

- Chest X-ray, which confirmed that the endotracheal tube was *in situ*. It also showed widening of the mediastinum (Figs 1 and 2)
- Urgent computerised axial tomography (CAT) scan of the chest (Figs 5 - 9)
- Blood gas analysis, which documented respiratory acidosis.
- Additional haematological tests (urea and electrolytes, full blood count, liver function, blood cultures and C-reactive protein) were mostly normal, with two exceptions – a high C-reactive protein of 172 ml/L and *Klebsiella pneumoniae* and *Candida fumata* were cultured from blood. The infections were treated appropriately.

**Fig. 3:**
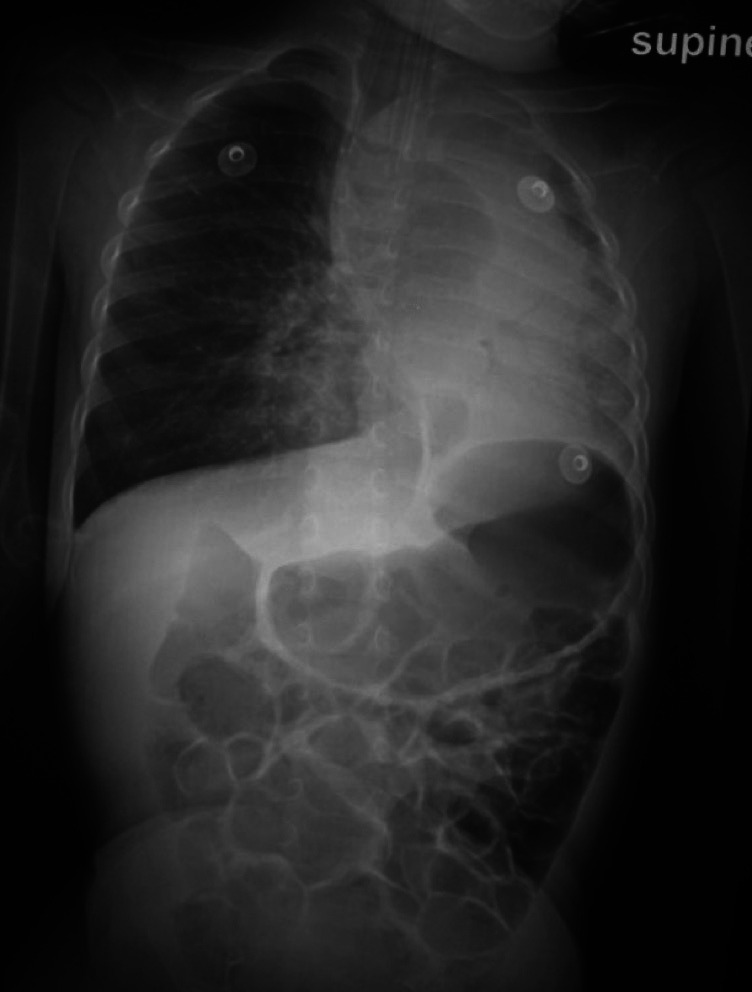
Superior mediastinal mass, collapsed left lung showing mediastinal shift to left. Right lung hyperinflation and herniation to the left.

**Fig. 4:**
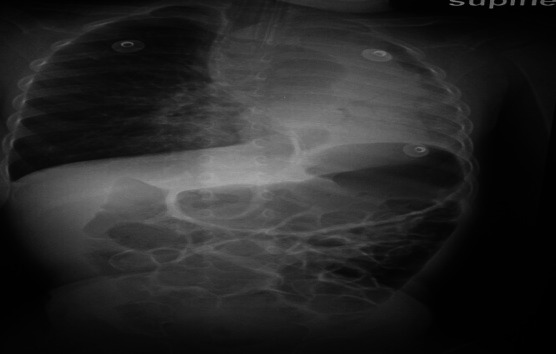
Superior mediastinal mass, collapsed left lung showing mediastinal shift to left. Right lung hyperinflation and herniation to the left.

**Fig. 5:**
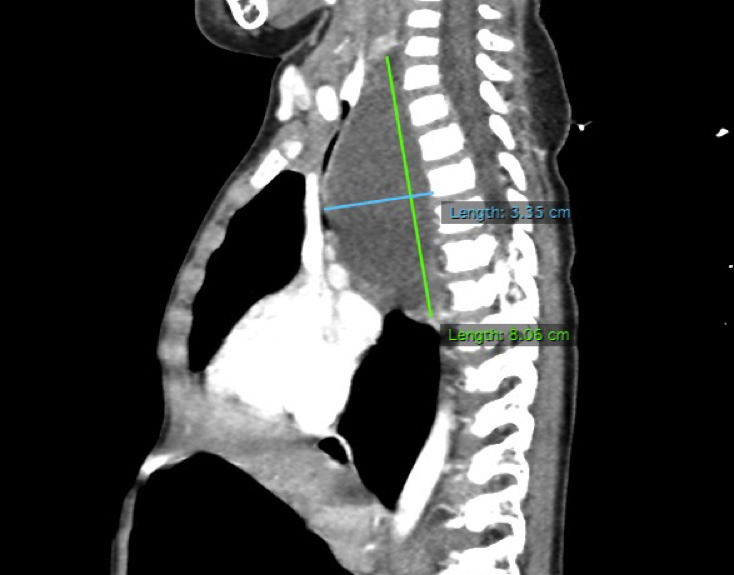
Middle mediastinal cystic mass lesion. Displacing and compressing the airway and the oesphagus.

**Fig. 6:**
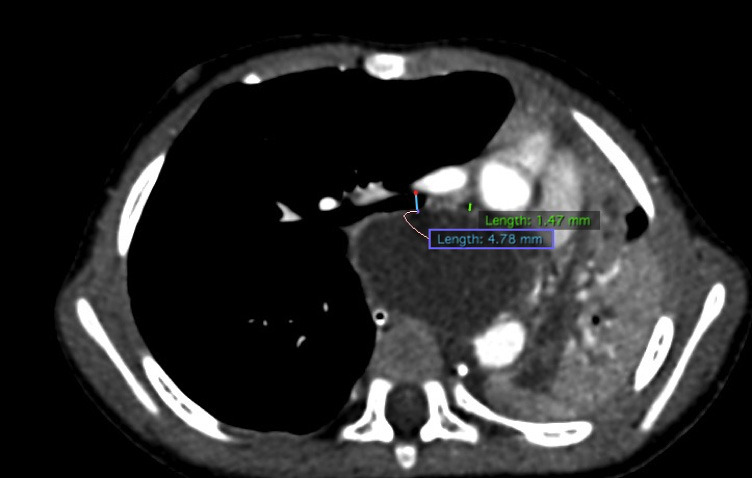
Atresia of left bronchus due to chronic mass effect.

**Fig. 7:**
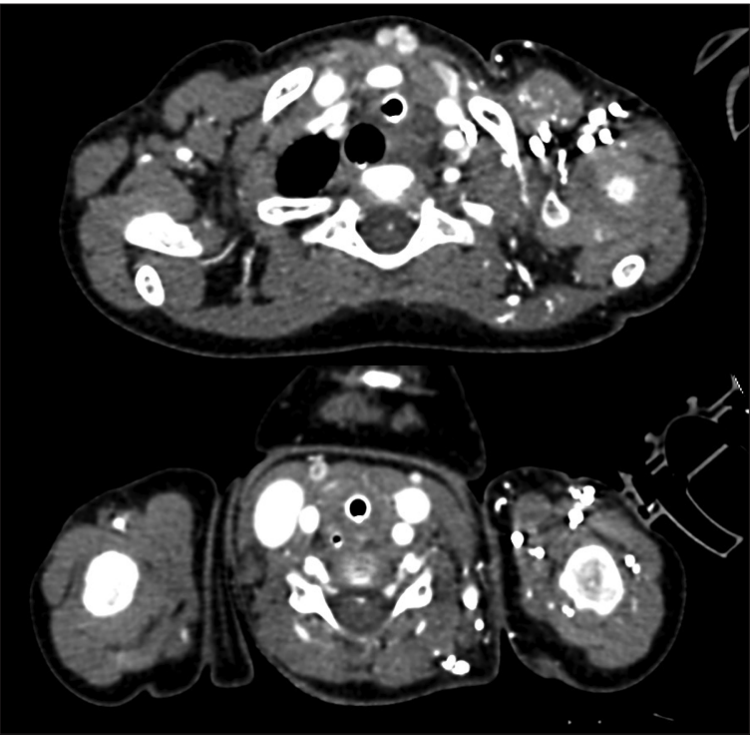
Prominent multiple veins noted on the anterior chest wall, neck and left shoulder with associated prominent right jugular vein from raised jugular venous pressure from compressed superior vena cava. Proximal oesophageal dilation with nasogastric tube *in situ* from proximal oesophageal mass effect.

**Fig. 8:**
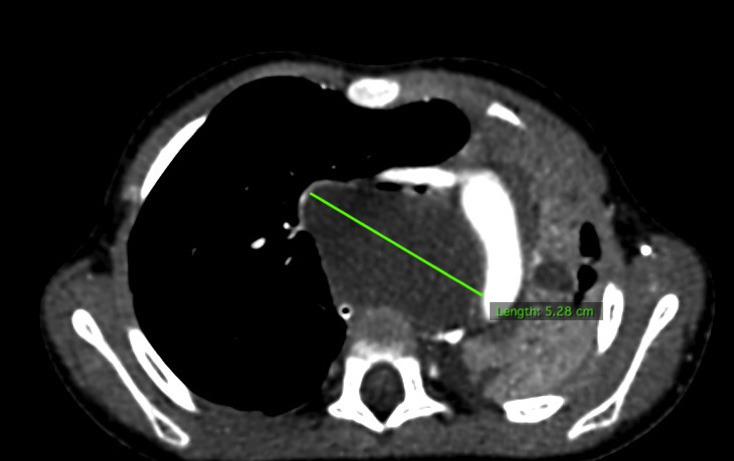
Middle mediastinal cystic mass with mass effect on the superior vena cava and trachea and with associated anterior displacement of the anterior mediastinum, left lung collapse and right lung compensatory hyperinflation.

**Fig. 9:**
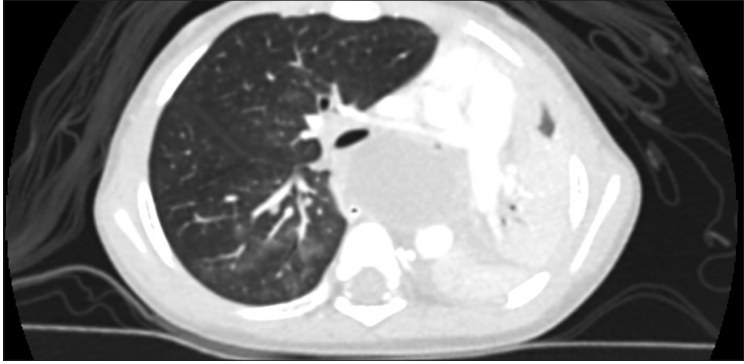
Right lung hyperinflation with multiple diffuse patchy infiltrates suggestive of infection and early consolidation.

The differential diagnosis at this point included tuberculous adenitis, lymphoma, bronchogenic cyst and thoracic neuroblastoma.

The mass was deemed to be exerting pressure on the oesophagus because, despite the insertion of a nasogastric tube, the child continued to vomit feeds.

The cardiothoracic surgical team was consulted and a diagnosis of a bronchogenic cyst was made. A thoracotomy was performed on day four of admission. Intraoperative findings were consistent with an intrathoracic cyst. A cystectomy was performed and an infected cyst was removed. Ventilation of the patient in PICU was continued postoperatively. The postoperative course was eventful – a new fungal sepsis was identified and managed. A surgical relook procedure and bronchoscopy were performed and the intraoperative findings did not show any collections or puss in the excised cystic area. Bronchoscopy revealed a left bronchomalacia, with thick secretions (Fig. 12).

**Fig. 10:**
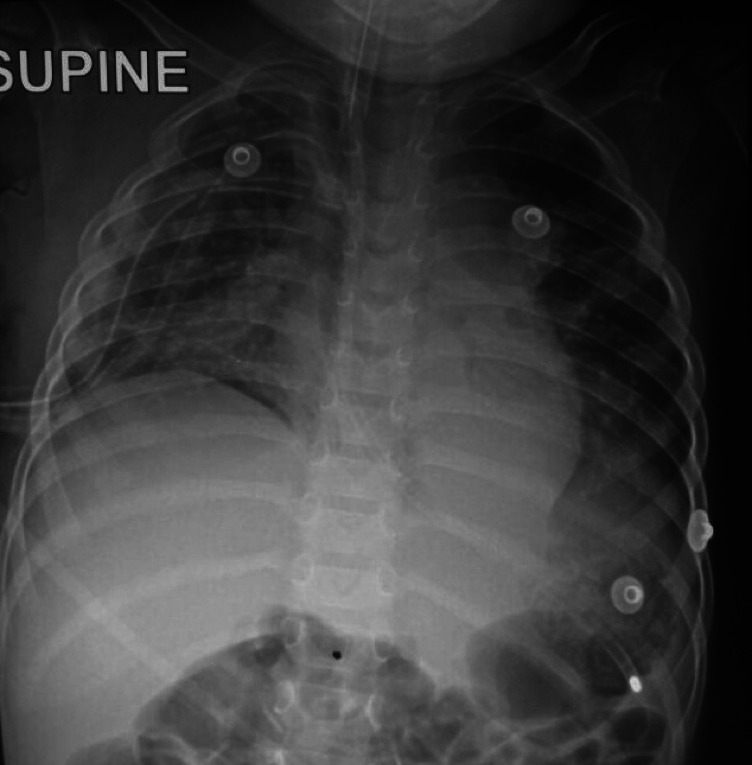
Postoperative chest X-rays. Endotracheal tube, nasogadtric tube, and intercostal drain *in situ*. Malaligned right upper ribs, right lung contusions. Superior mediastinum less dense and reduced. Left lung reinflated. Right lung normal volume.

**Fig. 11:**
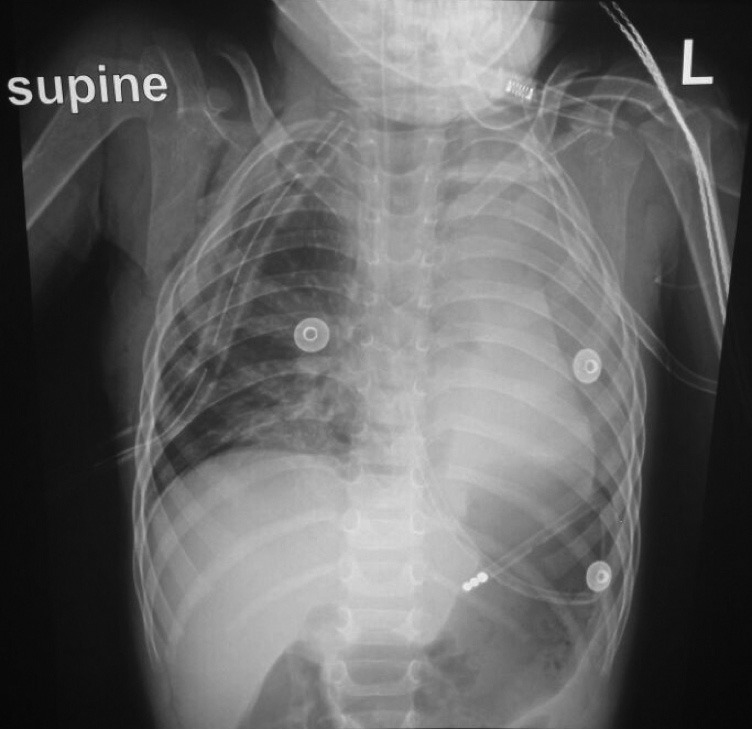
Endotracheal tube, nasogadtric tube, and intercostal drain *in situ*. Malaligned right upper ribs, right lung contusions. Superior mediastinum less dense and reduced. Left lung was reinflated, while the right lung had a normal volume.

**Fig. 12:**
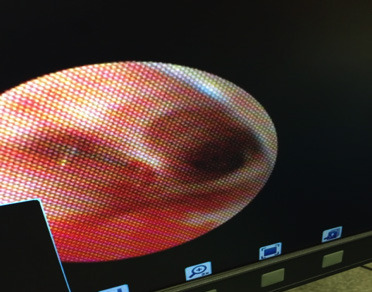
Bronchoscopy showing narrowing of the left main bronchus.

The patient was successfully ventilated for thirty days in PICU. At the time of submission of this manuscript for publication, the patient was well and not receiving any treatment for asthma. Post discharge control chest X-rays and CAT scan of the chest were normal.

## Histology of the cyst

Macroscopically, the specimen consisted of an opened cyst that measured 70 × 45 mm. Microscopic examination of the cyst showed a fibromuscular wall and a respiratory epithelium lining, with areas of squamous metaplasia. Cartilage was focally present in the wall. The cyst was multilocular with acute on chronic inflammation and destruction of epithelial lining and was identified as a mediastinal BC.

## Discussion

Although BCs are relatively rare, they are the most common cysts in the mediastinum. ^[1]^ Serious and life-threatening events can occur due to compression of major airways, severe infections, haemorrhages, malignant transformation, fistula formation, superior vena caval syndrome and rupture.^[2]^ Symptoms vary with age at presentation and up to a large extent depend upon size and location of the cyst. Most cysts are asymptomatic at an early age. Symptoms are related to the location and site of the cysts. In children, symptoms occur as a result of compression of the trachea, main bronchus and oesophagus.^[3]^

Chest X-ray (CXR) findings are nonspecific, but do suggest a possible mediastinal mass;^[4]^ however, CXR is the first line of investigations.^[4]^ CAT scan of the chest is useful in localising these cysts while also helping with the diagnosis and decision of surgical treatment.^[5]^ Treatment strategy of asymptomatic BCs is controversial, the consensus is that all BCs whether asymptomatic, symptomatic and those with complications should be treated surgically. ^[5]^ Some surgeons perform surgical intervention in all asymptomatic patents in order to establish diagnosis and to prevent complications.^[4]^

Foregut cysts are classified as BCs, which can be mediastinal or intrapulmonary and represent many of bronchopulmonary malformations.^[5]^ Foregut cysts arise from an abnormal budding of primitive foregut before the 16th week of gestation. They can be classified into enteral or BCs.^[3]^ The walls are made up of tissue similar to that of normal bronchial tree, including cartilages, elastic tissue, mucous glands and smooth muscle, usually solitary and lined by cuboidal or columnar ciliated epithelial cells.^[3]^

It is reported that the majority of BCs are located in the mediastinum, usually in close relationship to trachea and major conducting airways. ^[6]^ Although the majority of BCs are located inside the mediastinum, a series of 29 cases reported by Kosar *et al*. ^[5]^ found a majority of 78.4% in the intrapulmonary location.^[5]^ This they describe as somewhat controversial to the literature.

Intrapulmonary BCs are commonly located in the lower lobes of the lungs.^[6]^ A study by Chang *et al*. ^[6]^ confirmed this location of intrapulmonary BCs.In their study the most common location was the subpleural region of the lower lobe (55%), followed by the midlung (30%) and perihilar region (15%).^[6]^

Jiang *et al*. ^[7]^ confirmed in their case series that mediastinal BCs are observed more often in adults than children, 67% of their cases occurring in adults. A minority of BCs is asymptomatic.^[2]^ This may lead to productive cough, dyspnoea, chest pain, haemoptysis, fever. ^[2]^ Wheezing can occur due to compression of the airways and the patient can have digestive tract symptoms. Haemorrhages, recurrent Infections occur later in life and pleural perforations are possible.

Serious complications from BCs are rare, but can include superior vena caval syndrome, tracheal compression, pneumothorax, pleurisy and recurrent chest infections.^[4]^ There is risk of malignant degeneration to rhabdomyosarcoma, adenocarcinoma, and malignant melanoma.^[8]^ Bronchomalacia is one of the complications post removal of the cyst secondary to the compression of the mass to the bronchus. It can be associated with tracheobronchomalacia with the affected bronchus lacking rigidity because of insufficient cartilages or extrinsic compression.^[9]^ Acquired or secondary bronchomalacia can result from extrinsic compression of the bronchus.^[9]^ Flexible bronchoscopy performed by an experienced bronchoscopist is the gold standard in establishing the diagnosis of bronchomalacia.

Differential diagnosis of BCs includes lung abscess, hydatid cyst, infected bullae, lung sequestration, lobar emphysema, lymphoma, Neuroblastoma and tuberculous adenitis. Clinical findings and plain radiographs may be sufficient to make diagnosis, but confirmation with computed tomography scanning may help to establish nature of the lesion and further management.^[8]^ Barium swallows are sufficient for the diagnosis in 80% of cases at all ages. Computed axial tomography is accurate in determining the anatomy of mediastinal mass.^[2]^

Definitive diagnosis can only be confirmed by histopathological examination following surgical operation.^[5]^ For asymptomatic BCs treatment option remains controversial but general consensuses are that all BCs should be treated surgically even if asymptomatic, since the majority will ultimately become symptomatic or develop complications later on in life.^[5]^ Complete surgical resections for all symptomatic patient is recommended.^[10]^ This is because complete surgical resection is the only definite and radical management of mediasternal bronchogenic cyts, allows symptom suppression and achievement of a histological diagnosis.^[11]^ Histological findings in BCs are mostly the presence of ciliated columnar epithelial cells, smooth muscles bronchial gland, cartilage and even lung alveoli along the wall of the cyst.^[12]^

Other treatment options are transparietal, transbronchial or mediastinoscopic puncture and aspiration which are exceptionally preferred as temporal measures in case of acute compression in selected cases.^[7]^ Thoracosopic resection has major advantages that include less pain, better cosmetic and decreased risk of rib fusion. Video-assisted thoracic surgery is reported to be safe, convenient and practical with minimal morbidity. It is especially better in those who do not prefer thoracotomy.^[4]^ Lobectomy is the standard procedure as the cyst is often surrounded by areas of atelectasis and pneumonia for intrapulmonary cysts.^[5]^

## Conclusion

The persistently wheezing child and infant present a diagnostic and therapeutic dilemma to paediatricians.^[9]^ Patients who present with signs that mimic asthma and do not respond to the correct treatment for asthma need further investigations. In our case study the patient had more or recurrent chest infections and did not respond well to the asthma medications as prescribed by the general practioner. Simple chest X-rays and clinical assessment can provide more information to the clinician. Most of them are asymptomatic but can cause compressive symptoms or infections and complications.^[13]^ Surgical excision is indicated to enable definitive pathological diagnosis and to prevent complications and potential risk of malignant transformation in these patients.^[3]^
